# Dose–response modeling in mental health using stein‐like estimators with instrumental variables

**DOI:** 10.1002/sim.7265

**Published:** 2017-02-21

**Authors:** Cedric E. Ginestet, Richard Emsley, Sabine Landau

**Affiliations:** ^1^ Biostatistics Department, Institute of Psychiatry, Psychology and NeuroscienceKing's College LondonLondonU.K.; ^2^ Centre for Biostatistics, Institute of Population HealthUniversity of ManchesterManchesterU.K.; ^3^ MRC North West Hub for Trials Methodology ResearchLiverpoolU.K.

**Keywords:** ordinary least squares, two‐stage least squares, affine combination, stein estimators, mean squared error

## Abstract

A mental health trial is analyzed using a dose–response model, in which the number of sessions attended by the patients is deemed indicative of the dose of psychotherapeutic treatment. Here, the parameter of interest is the difference in causal treatment effects between the subpopulations that take part in different numbers of therapy sessions. For this data set, interactions between random treatment allocation and prognostic baseline variables provide the requisite instrumental variables. While the corresponding two‐stage least squares (TSLS) estimator tends to have smaller bias than the ordinary least squares (OLS) estimator; the TSLS suffers from larger variance. It is therefore appealing to combine the desirable properties of the OLS and TSLS estimators. Such a trade‐off is achieved through an affine combination of these two estimators, using mean squared error as a criterion. This produces the semi‐parametric Stein‐like (SPSL) estimator as introduced by Judge and Mittelhammer (2004). The SPSL estimator is used in conjunction with multiple imputation with chained equations, to provide an estimator that can exploit all available information. Simulated data are also generated to illustrate the superiority of the SPSL estimator over its OLS and TSLS counterparts. A package entitled SteinIV implementing these methods has been made available through the R platform. © 2017 The Authors. *Statistics in Medicine* Published by John Wiley & Sons Ltd.

## 1 Introduction

The use of instrumental variables (IVs) techniques has become increasingly popular in mental health trials to estimate causal treatment effects. Firstly, patients' non‐adherence with the psychotherapeutic treatment under offer may lead to selection bias. In the presence of such protocol violations, IV methods have often been used to estimate the complier average causal effect (CACE) 1, 2. Secondly, mental health researchers are typically interested in understanding treatment effect heterogeneity due to differences in therapeutic experience 3. We refer to variables that measure heterogeneity of treatment as *process variables*. Typical examples are the number of sessions of therapy, and the therapeutic alliance or the fidelity of the treatment delivery. Importantly, therapeutic process variables are post‐randomization variables that might be predicted by prognostic baseline variables, leading to such variables becoming *endogenous* with respect to linear models for mental health outcomes. (A predictor is said to be *exogenous*, if it is not correlated with the error term in the model. Otherwise, it is referred to as an endogenous predictor.) This issue has been addressed through the use of IV methods 4, 5, 6.

While the asymptotic properties of IV estimators such as the two‐stage least squares (TSLS) are well understood; in practice, it is not always clear whether or not using an IV estimator over a simpler ordinary least squares (OLS) estimator is necessarily beneficial. Intuitively, because every IV is a random variable, its inclusion in the analysis tends to increase the variance of the resulting estimator. Variance inflation is inversely proportional to the predictive power of the IVs for explaining variability in the endogenous variable. Poor or *weak* instruments are variables that are weakly predictive of the endogenous variables in the analysis model. Thus, although the use of an IV estimator is likely to lead to a significant decrease in the bias of the OLS estimator, it will also yield a more variable estimator. Because the true value of the parameters of interest is unknown in practice, it is generally not possible to evaluate whether the benefit of using a given set of instruments outweighs the cost in variance of incorporating them into the analysis. In addition, the use of weak instruments can also lead to a substantial amount of finite sample bias. Indeed, the use of weak instruments has been studied by 7, and these authors have shown that the inclusion of instruments with only weak linear relationships with the endogenous variables tends to inflate the bias of the IV estimator, eventually producing an estimator as biased as the original OLS estimator.

In this paper, we address this issue by utilizing a semi‐parametric Stein‐like (SPSL) estimator, originally introduced by 8, which combines the OLS and TSLS estimators in an affine fashion. In this framework, a sample estimate of the mean squared error (MSE) of the estimators under consideration is constructed. Because the MSE can be decomposed into a bias and a variance component, it provides a natural criterion for combining the OLS and TSLS estimators. The shrinkage parameter weighting the relative contributions of the two candidate estimators is adaptive, in the sense that it depends on the properties of the data, and takes into account the strength of the instruments. The idea of combining the OLS and TSLS estimators has been previously discussed in the literature 9. In particular, 10 has proposed an ‘almost unbiased estimator’ for simultaneous equations systems, which strikes a balance between two different *k*‐class estimators by weighting their relative contributions using the sample size and the number of variables in the model. Moreover, 9 has given an interpretation of the limited information maximum likelihood estimator as a combination estimator, which relies on a weighting of the OLS and TSLS estimators. Such combined estimators, however, do not attempt to estimate the respective contributions of each estimator using the data, as was performed by 8 and 11. An information‐theoretical argument is also used by 12 to justify this approach.

The main contribution of this article is to demonstrate the utility of the SPSL in analyzing mental health trials. We here describe a psychotherapeutic intervention using a dose–response model. In this study, the patients differ in the number of sessions of psychotherapy that they have received. We wish to know how the effect of treatment changes as the dose of therapy increases. Treatment dosage is here defined as the number of therapy sessions that a patient would attend if therapy had been offered. As most data sets in medical research, this application contains missing data, and the SPSL estimator must therefore be adapted to ensure that the results are valid under a missing at random (MAR) data‐generating mechanism. We thus use multiple imputation with chained equations (MICE) methods and compute the resulting standard errors (SEs) for the OLS, TSLS, and SPSL estimators. This article therefore provides the first detailed applications of the SPSL estimator to mental health trials.

The paper is organized as follows. In the next section, we describe the main trial data set and the causal parameters of interest. In Section 3, we recall the assumptions behind OLS and TSLS estimation and then describe the SPSL framework of 8. In Section 4, the theoretical properties of the SPSL are assessed using a range of different simulated data sets. The proposed methods are then applied to address dose–response questions in the trial of interest, in Section 5. The proofs of the two main propositions in this paper, as well as some further details about the simulations, have been deferred to the Appendix.

## 2 Dose–response models in mental health

In trials of psychological therapies, it is often of interest not only to establish whether the intervention is effective in the target population but also to describe the therapeutic processes that need to take place to enhance their efficacy. Therapists explain the absence of a therapeutic effect by important therapeutic processes such as the receipt of a sufficient amount of therapy or the establishment of an alliance with the therapist not having taken place. Thus, standard intention‐to‐treat analyses of trials of mental health interventions are increasingly accompanied by further explanatory analyses aimed at assessing such hypothesized treatment effect heterogeneity empirically 1, 2, 5. Here, we focus on treatment effect modification by the dose of therapy, as measured by the number of therapy session received.

Importantly, such explanatory aims bring with them their own statistical challenges – namely, the variables whose effects we are trying to estimate are endogenous in the model for the response. As we will show, such endogeneity leads to bias in the OLS estimator, while an IV approach might be able to avoid bias albeit at the cost of a loss in precision. We will later propose an estimator that optimally combines these two approaches. But before going into the estimation of effects of endogenous variables, we first provide a motivating trial example, clarify the parameters that capture treatment effect modification by process variables, and show that these parameters can indeed be represented by effects of endogenous dose variables in a linear model for the response.

### 2.1 The Study of Cognitive Re‐alignment Theory in Early Schizophrenia trial

We re‐analyze a mental health trial that has previously used standard IV methods to investigate treatment effect heterogeneity due to differences in therapeutic experience. Specifically, we focus on a trial investigating dose–response research questions. Here, the variable that is hypothesized to modify the causal effect of therapy, is the number of sessions of therapy that a patient would attend, if she had been offered a course of therapy. What we can observe for each patient is the endogenous variable: ‘number of sessions received’. Hence, there is a need to employ IV methods in order to avoid bias. In this trial, the focus of the research is on the modification of this dose–response relationship by therapeutic alliance.

The Study of Cognitive Re‐alignment Theory in Early Schizophrenia (SoCRATES) is a clinical trial investigating the effect of cognitive behavioral therapy or supportive counseling for individuals in addition to treatment‐as‐usual having suffered a first or second acute episode of schizophrenia 13. For simplicity, the two psychological therapy arms were here combined to form a group of 104 subjects. These psychological therapies were contrasted with TAU, which was here defined as routine hospitalization following an acute episode of schizophrenia. The TAU arm comprised 103 subjects. Individuals did not have access to these specific psychological therapies outside the trial. Thus, all trial participants allocated to the TAU arm did not receive any sessions of the respective psychological therapies, and thus, there was no contamination. However, there was non‐adherence in the active arm. That is, patients being offered treatment may have received different doses of therapy. In the most extreme case, participants did not take part in any psychotherapy sessions and thus effectively received TAU.

The clinical outcomes of the study included the subjects' scores on the Positive And Negative Syndrome Scale (PANSS). PANSS were administered at baseline to a total of 207 subjects, denoted by PANSS(0), and at an 18‐month follow‐up, denoted by PANSS(18). The data analyzed here constitute a subsample of the full data set, as delineated by Emsley *et al*. 14. The dose of psychological therapy was captured by the number of sessions that the patient took part in. In addition, psychotherapeutic alliance was measured using the short 12‐item patient‐completed version of the California Therapeutic Alliance Scale (CALPAS). Note that this is an interval psychometric scale. The scale cannot determine the absence of therapeutic alliance and only measures differences in the degree of alliance. For convenience, CALPAS scores were thus rescaled such that scores ranged from −7 to 0, with larger scores denoting greater psychotherapeutic alliance and a value of zero indicating the best therapeutic alliance achieved in this trial. A number of further clinical and demographic variables were measured at baseline (pre‐randomization), described in the next section. The SoCRATES data set contained a substantial amount of missing data, with 54 cases missing between one and five values, and 48 subjects for whom PANSS(18) was not available.

Several authors 4, 15, 16 have previously conducted analyses of the SoCRATES trial in order to understand how the perceived alliance of the patients with their therapist influences the relationship between the number of sessions received and the PANSS(18) outcome. We here replicate their analyses and expand them, in order to demonstrate the usefulness of the use of the SPSL estimator in this context.

### 2.2 Treatment effect modification by process variables

We here follow Rubin's causal model 13, which provides a framework for defining causal effects. We use the following notation for our trial, in which an active condition is compared with a control condition:

- *R* is *treatment offer*, to which the participant is randomly assigned (with *r*=0 for the control arm and *r*=1 for the active arm). In the SoCRATES trial, *R*=1 for those offered psychological therapy and *R*=0 for those offered TAU. By contrast, *T* is *treatment receipt*, defined as receiving at least one session of psychological therapy; *T*=1 if *S*>0. If *T*=1, then *R*=1, because of trial participants having no access to psychological therapy outside the trial.
- *Y*(*T*=*t*) is the *potential outcome under treatment* *t*. There are two potential outcomes, *Y*(*T*=1) and *Y*(*T*=0); only one of which can be observed for a given participant. The contrast Δ:=*Y*(*T*=1)−*Y*(*T*=0) then denotes an individual's causal treatment effect (ITE). Importantly, ITEs can vary between individuals. We will also utilize potential outcomes under treatment offer, denoted by *Y*(*R*=1) and *Y*(*R*=0), which potential outcome we refer to will be made explicit in these instances.
- *S*(1):=*S*(*R*=1) is the *number of sessions* that an individual would have taken part in, had they been offered the active condition. In the SoCRATES data set, this potential outcome is observed for the psychological therapies arm; it is missing for the control arm. Analogously, *S*(0):=*S*(*R*=0) is the number of therapy sessions that an individual would take part in, had they been offered the control condition. In SoCRATES, this potential outcome is *S*(0)=0 for all trial participants because they have no access to the active condition. The observed number of sessions, *S*, is related to the potential number of sessions via the equation *S*=*R* *S*(1). We refer to trial participants with *S*(0)=0 and *S*(1)>0 as ‘compliers’ and those with *S*(0)=0 and *S*(1)=0 as ‘never‐takers’. The subpopulation of compliers can be further divided into ‘one‐session takers’, satisfying *S*(0)=0 and *S*(1)=1, ‘two‐session takers’, satisfying *S*(0)=0 and *S*(1)=2, and so forth.
- *A*(1):=*A*(*T*=1) is the alliance (rescaled CALPAS score) that a participant would be able to build with the therapist across their sessions if they were to receive therapy. Such a potential outcome can only be defined for compliers (i.e., when *S*(1)>0). However, the product *S*(1)*A*(1) is defined for the whole sample. Variable *A* denotes the observed alliance score. Alliance cannot be observed for those who are not offered therapy or are offered but do not comply, and thus, the score is missing for such trial participants (i.e., when *S*=0). However, the product *S* *A* can be fully observed and is related to potential outcomes via the equation *S* *A*=*R* *S*(1)*A*(1).
- Finally, *B* denotes the *baseline outcome measure*, PANSS(0), and *X*_*j*_'s collectively refer to other *observed covariates*, including years of education and duration of untreated psychosis (DUP) in years, as well as two dummy variables that allow for differences between the three different centers.

We here restrict our attention to trials without contamination and study the effect of dose of therapy when offered, that is, the effect of *S*(*R*=1). We make the following causal assumptions:

1 . (C1) *No contamination:* *S*(*R*=0)=0.
2 . (C2) *Linear dose–response model:*
  - $Y(R=0)=\mu_{1}+\beta_{B}B+\sum_{j=1}^{k}\beta_{j}X_{j}+\tau$;
  - *Y*(*R*=1)=*Y*(*R*=0)+*β*_*S*_ *S*(*R*=1)+*β*_*SA*_ *S*(*R*=1)*A*(*T*=1)+*ν*.
3 . (C3) *No effect of treatment offer in never‐takers (exclusion restriction):* E[ν|S(R=1)=0,A(T=1)=a]=0, for every a∈(−∞,0].
4 . (C4) *No unaccounted variability in average treatment effects in compliers:* E[ν|S(R=1)=s,A(T=1)=a]=0, for every *s*∈{1,2,…}, and a∈(−∞,0].
5 . (C5) *Exchangeability of treatment offer:* *Y*(*R*=1),*Y*(*R*=0)⊥*R*.

Assumption (C1) implies that our target population does not contain any so‐called ‘always‐takers’ (here defined by *S*(*R*=0)>0 and *S*(*R*=1)>0) nor any ‘defiers’ (here defined by *S*(*R*=0)>0 and *S*(*R*=1)=0). A crucial consequence of this assumption is the following relationship between the observable and potential outcomes: *S*=*R* *S*(1)=*S* and *S* *A*=*S* *A*(1)=*R* *S*(1)*A*(1).

We seek to understand how being able to take part in more sessions, *S*(1), changes the efficacy of the therapy and how this dose–response relationship is modified by the therapeutic alliance a participant is able to build when receiving therapy, *A*(1). Assumption (C2) employs a linear model to describe these relationships. The parameter *β*_*S*_ describes the change in the potential outcome under treatment offer, *Y*(*R*=1), per one extra session taken part in for those who can build an optimal alliance with the therapist. In SoCRATES where improvements are reflected by a lowering of PANSS(18), we anticipated this parameter to be negative. The second parameter *β*_*SA*_ models the modification of this relationship by alliance. Specifically, this parameter reflects the change in the session effect as alliance increases by one point.

The residual term *ν* represents unaccounted variability in the causal treatment offer effect *Y*(*R*=1)−*Y*(*R*=0), in a subpopulation indexed by *s* and *a*. Causal assumptions (C3) and (C4) are concerned with this variability. Assumption (C3) states that for never‐takers (*S*(1)=0), the expectation of this residual is zero; that is, to say the average treatment offer effect in never‐takers is zero. This assumption is conventionally referred to as the exclusion restriction assumption in trials. Assumption (C4) is concerned with this variability in the compliers (*S*(1)=*s* with *s*>0). For compliers, *Y*(*R*=1)−*Y*(*R*=0)=*Y*(*T*=1)−*Y*(*T*=0), and thus, we are making an assumption regarding the variability of the ITEs. We assume that there is no unaccounted variability in the *average* treatment effect across complier subpopulations indexed by *s* and *a*. That is to say, our linear model has accounted for all the heterogeneity in average treatment effects across sessions and alliance scores. For example, this implies that for any *A*(1)=*a*, the relationship between average treatment effects and the number of sessions a participant would take part in is truly linear. Finally, assumption (C5) states that treatment offer is ignorable. Exchangeability of treatment offer *R* is ensured in trials because of randomization.

We can now formally express the average causal treatment effect in the subpopulation of patients who would take part in *s*>0 therapy sessions and would achieve an alliance score of *a*, as a linear function of these scores. Specifically, utilizing (C1), (C2), and (C4), we can then write the local average treatment effect, ${\text{LATE}}_{s,a}:=E[\Delta |S(R=1)=s,A(T=1)=a]$, for compliers as follows:

(1) $$\begin{array}{ccc} {\text{LATE}}_{s,a} & =E[\beta_{S}S(1)+\beta_{SA}S(1)A(1)+\nu |S(1)=s,A(1)=a] & \\ & =E[\beta_{S}s+\beta_{SA}sa|S(1)=s,A(1)=a]\\ & =\beta_{S}s+\beta_{SA}sa, \end{array}$$

for every *s*∈{1,2,…} and a∈(−∞,0], where the first equality is an application of (C2) and the second equality follows from the linearity of the expectation, as well as (C4) for eliminating the error term.

It is now easy to see that for compliers, the parameters, *β*_*S*_ and *β*_*SA*_, describe the modification of the causal estimand, LATE_*s*,*a*_, by the process variables, *S*(*R*=1) and *A*(*T*=1). For those who achieve the maximum alliance with the therapist (i.e., *a*=0), the change for every extra session is given by LATE_*s*+1,0_−LATE_*s*,0_=*β*_*S*_(*s*+1)−*β*_*S*_ *s*=*β*_*s*_, and when reducing the alliance score by one point, this relationship is modified to become LATE_*s*+1,−1_−LATE_*s*,−1_=*β*_*S*_(*s*+1)−*β*_*SA*_(*s*+1)−(*β*_*S*_ *s*−*β*_*SA*_ *s*)=*β*_*S*_−*β*_*SA*_. Finally, the LATEs can be used to define the CACE as follows: CACE:=E[Y(T=1)−Y(T=0)|S(R=1)>0].

### 2.3 Correspondence with linear model

Utilizing assumptions (C1) and (C2), we obtain the following linear model. The full details of this derivation are provided in Appendix A.1.

(2) $$Y=\mu_{1}+\beta_{B}B+\overset{k}{\underset{j=1}{\sum }}\beta_{j}X_{j}+\beta_{S}S+\beta_{SA}SA.$$

Thus, the parameters of interest correspond to the effects of the explanatory variables, *S* and *S* *A*, in a linear model for *Y*. The combined error term of the linear model may be denoted by *ε*:=*τ*+*R* *ν*, with E[ε|S,SA]=E[τ|S,SA]≠0. It is then apparent that explanatory variables, *S* and *S* *A*, may be endogenous. The covariance between the number of therapy sessions received, *S*, and the noise term *ε*, for instance, could be due to an omitted common cause. The same argument holds for the explanatory variable, *S* *A*.

Because of the exclusion restriction stated in assumption (C3), and to the exchangeability stated in (C5), treatment offer, *R*, does not have a direct effect on the outcome (for more details, see Appendix A), and moreover, *R* and the outcome variable do not share a common cause. Therefore, this provides us with the opportunity of using *R* as an IV; see Section 3. Note also that in the presence of treatment effect variability within subpopulations (i.e., Var(ν)>0), the variance of the model's error term Var(ε)=Var(τ+Rν) might be increased for those being offered therapy (*R*=1), with the increase possibly depending on the subpopulation.

Thus, we are interested in estimating the regression coefficient of the explanatory variables *S* and *S* *A* in the model for PANNS(18). Both of these explanatory variables are endogenous in this model, whereas the remaining covariates are exogenous. We therefore require at least two IVs, in order to estimate these effects without bias. In line with 3, we assume the following bivariate model for these variables, which includes a set of interaction terms between treatment allocation, *T*, and the baseline variables:

(3) $$\left(\begin{array}{c} S\\ SA \end{array}\right)=\mu_{2}+\xi_{B}B+\overset{m}{\underset{j=1}{\sum }}\xi_{j}X_{j}+\left(\right.\theta_{T}+\theta_{B}B+\overset{m}{\underset{j=1}{\sum }}\theta_{j}X_{j})R+\delta ,$$

in which ***μ***_2_, the ***ξ***'s, and the ***θ***'s are unknown parameters. The equations in ((2)) and ((3)) will be used within a TSLS approach in order to estimate the two parameters describing treatment effect heterogeneity across different subpopulations of participants, characterized by the number of sessions that a participant would be likely to take if offered therapy and the degree of alliance that they would likely build with the therapist.

## 3 Combining ordinary least squares and two‐stage least squares estimators

We now turn to a formal description of the Stein‐like estimator, which combines the OLS and TSLS estimators in an affine fashion.

### 3.1 Model assumptions

A two‐stage model with IVs and additional covariates is used for our data set. (We here adopt the econometrics convention of referring to the model for the endogenous variables and the model for the outcome variable, as the first‐stage and second‐stage models, respectively.) The second‐stage model is a model for the clinical outcomes that contains regression coefficients representing our parameters of interest. The outcome measure is denoted by the column vector **y**, which stands for PANSS(18) in the SoCRATES trial. The design matrix **X** has *k* columns, which represent the predictors in the model. These predictors are partitioned into *k*_1_ *endogenous* variables denoted by **X**_1_ and *k*_2_ *exogenous* variables denoted by **X**_2_, such that **X**:=[**X**_1_ **X**_2_]. The outcome variable is modeled as follows:

(4) $$y=X_{1}\beta_{1}+X_{2}\beta_{2}+\varepsilon ,$$

where the parameters ***β***_1_ and ***β***_2_ are column vectors with respective dimensions *k*_1_ and *k*_2_ with *k*=*k*_1_+*k*_2_ and the error term ***ε*** has dimensions *n*×1. In the SoCRATES data set, **X**_1_ contains the number of sessions and the interaction between the number of sessions and alliance, as measured by the CALPAS scale, whereas **X**_2_ includes an intercept, two dummy variables for the different centers, years of education, DUP, and PANSS(0).

Through our choice of notation for **X**:=[**X**_1_ **X**_2_], equation ((4)) can be written more compactly as **y**=**X** ***β***+***ε***, in which $\beta :={\left[\beta_{1}^{\prime }\;\beta_{2}^{\prime }\right]}^{\prime }$. If we assume that the error terms are homoscedastic with respect to **X**, such that $E[\varepsilon_{i}^{2}|X]=\sigma^{2}$, for every *i*=1,…,*n*, and that the design matrix is full‐rank, such that $\text{rank}(X^{\prime }X)=k$, it then follows that the OLS estimator is well identified for this model and is given by $\tilde{\beta }:={\left(X^{\prime }X\right)}^{-1}(X^{\prime }y)$. The OLS estimator can be shown to be unbiased, if the variables in **X** are assumed to be exogenous. This assumption requires that $E[X^{\prime }\varepsilon ]=0$, or equivalently that $E[x_{i}^{\prime }\varepsilon_{i}]=0$, for every *i*=1,…,*n*, because the *ε*_*i*_'s are assumed to be identically distributed, and where each **x**_*i*_ denotes the i*th* row of **X**. When this is the case and the first moment of this estimator exists, the OLS estimator is asymptotically unbiased and consistent such that $E[\tilde{\beta }]=\beta +E[{\left(X^{\prime }X\right)}^{-1}(X^{\prime }\varepsilon )]$, and the second term cancels out, because of the exogeneity of **X**, as n→∞.

In the data set of interest, however, the variables in **X**_1_ cannot be assumed to be exogenous. Therefore, we will make use of a matrix of instruments, denoted **Z**_1_, of dimensions *n*×*l*_1_. These instruments are combined with the *k*_2_ exogenous variables from the second‐stage equation in order to produce the following first‐stage equation. Observe that this portion of the model is a multivariate multiple regression, because its outcome variable, **X**, is a matrix,

(5) $$X=Z_{1}\Gamma_{1}+X_{2}\Gamma_{2}+\delta .$$

**Figure 1:**
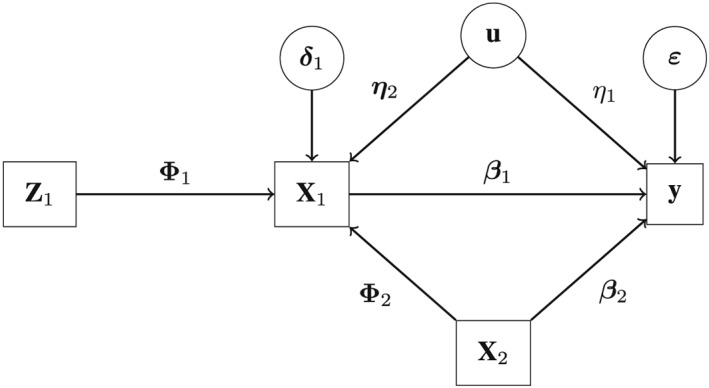
Graphical representation of the instrumental variable model described in equations ((4)) and ((5)), composed of a set of endogenous variables, **X**_1_, and a set of exogenous variables, **X**_2_. This graph corresponds to a two‐stage system of equations composed of **y**=**X**_1_ **β**_1_+**X**_2_ **β**_2_+**u** η_1_+**ε** and **X**_1_=**Z**_1_ **Φ**_1_+**X**_2_ **Φ**_2_+**u** **η**_2_+**δ**_1_, where **u** denotes a vector of unobserved confounders, while η_1_ and **η**_2_ represents its effect on **X**_1_ and **y**, respectively. The matrices of parameters **Φ**_1_,**Φ**_2_, and **δ**_1_ are of order l_1_×k_1_,k_2_×k_1_, and n×k_1_, respectively, and **η** is a vector of order 1×k_1_. (For convenience, we have here omitted the arrow linking **Z**_1_ and **X**_2_.)

Here, **Γ**_1_ and **Γ**_2_ are matrices of parameters of order *l*_1_×*k* and *k*_2_×*k*, respectively, with *l*:=*l*_1_+*k*_2_. Moreover, ***δ*** is a matrix of order *n*×*k* of error terms. A graphical representation of the two levels of the model in the presence of an unobserved confounder *U* is given in Figure 1. As for the OLS estimator, we can adopt the shorthands **Z**:=[**Z**_1_ **X**_2_] and $\Gamma :={\left[\Gamma_{1}^{\prime }\;\Gamma_{2}^{\prime }\right]}^{\prime }$, which are of order *n*×*l* and *l*×*k*, respectively. Equipped with these block matrices, the model in equation ((5)) can be rewritten in a more concise form as **X**=**Z** **Γ**+***δ***.

If, in addition, we assume that the instruments are exogenous with respect to the error term in equation ((4)), such that $E[Z^{\prime }\varepsilon ]=0$, and that the error term is homoscedastic with respect to **Z**, such that $E[\varepsilon_{i}^{2}|Z]=\sigma^{2}$ for every *i*=1,…,*n*, it then follows that we can construct an asymptotically unbiased and consistent estimator, assuming that the first moment exists. For such an estimator to be well identified, we also need to assume that **Z** is full‐rank such that $\text{rank}(E[Z^{\prime }Z])=l$ and moreover that $\text{rank}(E[Z^{\prime }X])=k$, as commonly carried out in econometrics [see 14, for details]. Under these assumptions, we can then recover the standard TSLS estimator formula, which is given by $\hat{\beta }:={\left({\hat{X}}^{\prime }\hat{X}\right)}^{-1}({\hat{X}}^{\prime }y)$, with $\hat{X}:=H_{z}X$ denoting the projection of the matrix of predictors onto the column space of **Z** and where $H_{z}:=Z{\left(Z^{\prime }Z\right)}^{-1}Z^{\prime }$ is the hat matrix of the multivariate regression in equation ((5)). It also follows that this model is well identified whenever there are at least as many instruments as endogenous variables – that is, when $l_{1}⩾k_{1}$, as in the data set at hand.

In summary, we have made the following set of *linear* assumptions. These assumptions should be combined with the assumptions made in Section 2. Firstly, the computation of the OLS requires the following two standard assumptions:

1 . (OLS‐1) *Homoscedastitity:* $E[\varepsilon^{2}|X]=\sigma^{2}$,
2 . (OLS‐2) *Identification:* $\text{rank}(E[X^{\prime }X])=k$.

Secondly, as commonly carried out in econometrics [:], the derivation of the TSLS requires the following

1 . (TSLS‐1) *Exogeneity:* $E[Z^{\prime }\varepsilon ]=0$,
2 . (TSLS‐2) *Homoscedasticity:* $E[\varepsilon^{2}|Z]=\sigma^{2}$,
3 . (TSLS‐3) *Identification:* $\text{rank}(E[Z^{\prime }Z])=l$, $\text{rank}(E[Z^{\prime }X])=k$;
4 . (TSLS‐4) *Relevance:* Cov(X,Z)≠I.

Observe that, despite the fact that condition (TSLS‐4) resembles condition (C4), these two conditions are not necessarily related. Under the aforementioned assumptions, the empirical variance of the OLS and TSLS estimators can be consistently estimated using the standard formulas $\tilde{V\text{ar}}(\tilde{\beta }):={\tilde{\sigma }}^{2}{\left(X^{\prime }X\right)}^{-1}$ and $\tilde{V\text{ar}}(\hat{\beta }):={\hat{\sigma }}^{2}{\left({\hat{X}}^{\prime }\hat{X}\right)}^{-1}$, with the sample residual sums of squares, ${\tilde{\sigma }}^{2}$ and ${\hat{\sigma }}^{2}$, being given by ${\left(y-X\tilde{\beta }\right)}^{\prime }(y-X\tilde{\beta })/(n-k)$ and ${\left(y-X\hat{\beta }\right)}^{\prime }(y-X\hat{\beta })/(n-k)$ for the sample residual sums of squares of the OLS and TSLS estimators, respectively. More remarkably, the bias of these two estimators can also be estimated from the data in a consistent fashion. Indeed, because the TSLS estimator is asymptotically unbiased, it is natural to use this estimator in order to quantify the bias of the OLS estimator. Therefore, one may approximate the squared bias of a candidate estimator, say ***β*** ^*†*^, as follows: $\hat{B{\text{ias}}^{2}}(\beta^{†}):=(\beta^{†}-\hat{\beta }){\left(\beta^{†}-\hat{\beta }\right)}^{\prime }$. Moreover, because both ***β*** ^*†*^ and $\hat{\beta }$ are consistent estimators of $E[\beta^{†}]$ and $E[\hat{\beta }]$, respectively, it then follows that $\hat{B{\text{ias}}^{2}}(\beta^{†})$ is a consistent estimator of the true squared bias of ***β*** ^*†*^. This particular choice of empirical bias estimate can be seen to be related to the Hausman test, commonly used in econometrics for testing whether or not some predictors of interest are exogenous 17. Indeed, if one were to estimate the bias of the OLS estimator using this particular method, we would obtain $\hat{B{\text{ias}}^{2}}(\hat{\beta })=(\hat{\beta }-\tilde{\beta }){\left(\hat{\beta }-\tilde{\beta }\right)}^{\prime }$, which exactly corresponds to the trace of the numerator of the Hausman test.

Combining this empirical estimate of the bias with the standard variance estimators, we can formalize a classical observation about the superiority of the TSLS estimator in terms of (estimated) bias and the superiority of the OLS estimator in terms of (estimated) variance. Results of this type have motivated the construction of combined estimators, such as the SPSL estimator introduced by 8. For completeness, a full proof of this result is provided in the Appendix.

Proposition 1 Under assumptions (OLS‐1), (OLS‐2), (TSLS‐1), (TSLS‐2), and (TSLS‐3), we have (i) $\hat{B{\text{ias}}^{2}}(\tilde{\beta })≽\hat{B{\text{ias}}^{2}}(\hat{\beta })$ and (ii) $\hat{V\text{ar}}(\tilde{\beta })≼\hat{V\text{ar}}(\hat{\beta })$, where ≽ and ≼ denote the positive semi‐definite order for *k*×*k* matrices.

### 3.2 Semi‐parametric Stein‐like estimator

In a series of publications, Judge and Mittelhamer have introduced the SPSL estimator and studied its asymptotic properties 8, 11, 12, 18, 19. The SPSL estimator is defined as an affine combination of an unbiased estimator, such as the TSLS, and another estimator, such as the OLS. In the notation adopted in the previous section, the SPSL estimator is thus defined as follows:

$$\bar{\beta }(\alpha ):=\alpha \hat{\beta }+(1-\alpha )\tilde{\beta },$$

for every α∈R. The *shrinkage parameter*, *α*, controls the respective contributions of the OLS and TSLS estimators. (Despite our choice of name, however, note that *α* needs not be bounded between 0 and 1.) This parameter is selected in order to minimize the trace of the theoretical MSE of the corresponding SPSL estimator,

$$\text{MSE}(\bar{\beta }(\alpha ))=E\left[\right.\left.\right.(\bar{\beta }(\alpha )-\beta ){\left(\bar{\beta }(\alpha )-\beta \right)}^{\prime }]=V\text{ar}(\bar{\beta }(\alpha ))+B{\text{ias}}^{2}(\bar{\beta }(\alpha )),$$

where $\beta \in R^{k}$ is the true parameter of interest and the MSE is a *k*×*k* matrix. It is particularly appealing to combine these two estimators, because the asymptotic unbiasedness of the TSLS estimator guarantees that the resulting SPSL is asymptotically unbiased. Thus, the MSE automatically strikes a trade‐off between the unbiasedness of the TSLS estimator and the efficiency of the OLS estimator. In particular, one should emphasize that although the SPSL trades off finite sample variance with finite sample bias, it retains asymptotic unbiasedness. Therefore, in the light of proposition 1, this criterion constitutes a natural choice for combining these two types of estimators.

The MSE of the SPSL estimator, $\text{MSE}(\alpha \hat{\beta }+(1-\alpha )\tilde{\beta })$, can be expressed as the weighted sum of the respective MSEs of the OLS and TSLS estimators, as well as a *cross squared error* (CSE) term between these two estimators. That is,

(6) $$\text{MSE}(\bar{\beta }(\alpha ))=\alpha^{2}\text{MSE}(\hat{\beta })+2\alpha (1-\alpha )\text{CSE}(\hat{\beta },\tilde{\beta })+{\left(1-\alpha \right)}^{2}\text{MSE}(\tilde{\beta }),$$

where the cross term is defined as follows: $\text{CSE}(\hat{\beta },\tilde{\beta }):=E[(\hat{\beta }-\beta ){\left(\tilde{\beta }-\beta \right)}^{\prime }]$. By analogy with the MSE, we can also decompose the CSE into a covariance term and a squared *cross‐bias* term, denoted $B{\text{ias}}^{2}(\hat{\beta },\tilde{\beta })$, such that $\text{CSE}(\hat{\beta },\tilde{\beta })=C\text{ov}(\hat{\beta },\tilde{\beta })+B{\text{ias}}^{2}(\hat{\beta },\tilde{\beta })$, where the squared cross‐bias term is $B{\text{ias}}^{2}(\hat{\beta },\tilde{\beta }):=(E[\hat{\beta }]-\beta ){\left(E[\tilde{\beta }]-\beta \right)}^{\prime }$.

The true (or theoretical) shrinkage parameter, *α*, is defined as the value that minimizes the trace of the theoretical MSE of the SPSL estimator. Note that we are here considering a sequence of parameters, *α*. Indeed, the shrinkage parameter will vary with different sample sizes. Therefore, for every *n*, the target shrinkage parameter is given by

(7) $$\alpha :=\underset{\alpha^{\prime }\in R}{\text{argmin}}\;\text{tr}\;\text{MSE}(\bar{\beta }(\alpha^{\prime })).$$

Crucially, this parameter is available in closed form, and it can also be shown to be unique, because the trace of the theoretical MSE of $\bar{\beta }$ is a convex function of *α*. This statement is made formal in the following proposition, which is proved using the aforementioned decomposition of the MSE of the SPSL estimator. The shrinkage parameter is only non‐unique when the square root of the trace of the MSEs of the OLS and TSLS estimators is identical. This quantity, denoted by (trMSE(***β*** ^*†*^))^1/2^ for every estimator ***β*** ^*†*^, will be referred to as the root mean squared error of ***β*** ^*†*^, in the sequel. A full detailed proof of this result, including a proof of the convexity of the criterion, is provided in the Appendix.

Proposition 2 For every *n*, the shrinkage parameter defined in equation ((7)) is given by

$$\alpha =\frac{\text{tr}(\text{MSE}(\hat{\beta })-\text{CSE}(\tilde{\beta },\hat{\beta }))}{\text{tr}(\text{MSE}(\hat{\beta })-2\text{CSE}(\tilde{\beta },\hat{\beta })+\text{MSE}(\tilde{\beta }))}.$$

Moreover, if the random vectors, $\tilde{\beta }$ and $\hat{\beta }$, are element‐wise squared‐integrable, then *α* is unique whenever the root mean squared errors of the OLS and TSLS estimators are not equal.

This shrinkage parameter can be estimated from the data by replacing the unknown theoretical quantities in proposition 2 with their sample estimates. Because, asymptotically, the estimated sample bias of the TSLS estimator is null, the formula for $\hat{\alpha }$ simplifies to

$$\hat{\alpha }=\frac{\text{tr}(\tilde{V\text{ar}}(\hat{\beta })-\tilde{\text{CSE}}(\tilde{\beta },\hat{\beta }))}{||\tilde{\beta }-\hat{\beta }{\left|\right|}^{2}},$$

where ||·|| denotes the *L*_2_‐norm on $R^{k}$, with respect to the *empirical* joint distribution of **y**,**X** **,** and **Z**, such that $\left||\tilde{\beta }-\hat{\beta }{\left|\right|}^{2}:=\tilde{E}[{\left(\tilde{\beta }-\hat{\beta }\right)}^{\prime }(\tilde{\beta }-\hat{\beta })\right]$. The empirical SPSL estimator can then be expressed in a familiar Stein‐like format 20, as a weighted deviation from the unbiased TSLS estimator, thereby justifying SPSL as a choice of name:

$$\bar{\beta }(\hat{\alpha })=\tilde{\beta }-\frac{\hat{\tau }}{||\hat{\beta }-\tilde{\beta }{\left|\right|}^{2}}(\hat{\beta }-\tilde{\beta }),$$

where $\hat{\tau }:=\text{tr}(\hat{V\text{ar}}(\hat{\beta })-\hat{\text{CSE}}(\hat{\beta },\tilde{\beta }))$. See also 19.

As before, if we assume that the random vectors, $\hat{\beta }$ and $\tilde{\beta }$, are well behaved, in the sense that they are element‐ wise squared‐integrable for every *n*, we can obtain a central limit theorem for the SPSL estimator, as was demonstrated by 19. From the definition of *α*, it also immediately follows that the SPSL estimator dominates both the OLS and TSLS estimators in quadratic risk.

## 4 Data simulations

We have carried out Monte Carlo simulations in order to evaluate the statistical properties of the SPSL estimator and to contrast them with those of the OLS and TSLS estimators for different degrees of endogeneity and different levels of instrument's strength.

### 4.1 Simulation model

Synthetic data sets were created for a two‐stage model with a dose process variable, as in the SoCRATES data set. This model contains the following variables: the outcome *Y*_*i*_, the baseline variable *B*_*i*_, the number of sessions attended by a given subject *S*_*i*_, and the experimental factor *R*_*i*_, as well as an unmeasured confounder *U*_*i*_. Then, for every subject, we have

(8) $$\begin{array}{ccc} Y_{i} & =\beta_{B}B_{i}+\beta_{S}S_{i}+\eta U_{i}+\varepsilon_{i} & \\ S_{i} & =\xi_{B}B_{i}+\xi_{R}R_{i}+\xi_{TB}R_{i}B_{i}+\eta U_{i}+\delta_{i}, \end{array}$$

in which the *ε*_*i*_'s and *δ*_*i*_'s are unknown error terms that are assumed to be independent of each other. Note that we are here treating the *ε*_*i*_'s as having the same variance. Throughout the simulations, we will be assuming that *B*_*i*_ and *U*_*i*_ are mutually independent and identically distributed according to a standard normal distribution (i.e., centered at zero, and with unit variance), whereas the variances of the error terms will be denoted by $\sigma_{\varepsilon }^{2}$ and $\sigma_{\delta }^{2}$, for *ε* and *δ*, respectively and the variance of the *B*_*i*_'s will be denoted by $\sigma_{B}^{2}$. The experimental factor, *R*_*i*_, is given a Bernoulli distribution with success probability *p*:=1/2. Consequently, the variance of the *R*_*i*_'s is $V\text{ar}(R_{i})=1/4$. In addition, we set the effect of the baseline variable, *B*_*i*_'s, to *β*_*B*_:=1/4 and its variance to $\sigma_{B}^{2}:=0.3$. The full details of the standardization of the parameters is given in Appendix C.1.

The simulation parameters are *η* and *κ*, which respectively control the amount of confounding and the strength of the instruments. With our chosen specification, we obtain the following relationships:

$$C\text{or}(S_{i},U_{i})=\eta \;\text{and}\;C\text{or}(S_{i},B_{i}+R_{i}+R_{i}B_{i})=\kappa .$$

Thus, *η* controls the *degree of endogeneity* of the *S*_*i*_'s, whereas *κ* controls the amount of covariance between *S*_*i*_'s and the combined instruments *B*_*i*_,*R*_*i*_, and *R*_*i*_ *B*_*i*_, such that *κ* can be interpreted as the *strength of the instruments*. We wish to keep the marginal variances of the *Y*_*i*_'s and *X*_*i*_'s at unity, while varying the values of *η* and *κ*. This is achieved by defining the variances of the error terms, *ε*_*i*_ and *δ*_*i*_, as functions of *η* and *κ*. In doing so, we simplify the interpretation of the parameter of interest, *β*_*S*_, which becomes a standardized regression coefficient. Throughout these simulations, the target parameter will take the value *β*_*S*_=1/4.

We evaluate the finite sample performance of the estimators of interest by comparing the Monte Carlo estimates of three different population parameters. For every candidate estimator, *β* ^*†*^, its Monte Carlo distribution is given by the following empirical distribution function: $\tilde{F}(b):=m^{-1}\sum_{t}I\{\beta_{t}^{†}⩽b\}$, where $I\{f_{t}\}$ denotes the indicator function taking a value of 1, if *f*_*t*_ is true, and 0 otherwise. For each simulation scenario, we draw *m*:=10^5^ realizations from the model in equation ((8)). The simulation scenarios were varied by selecting *η* to take the values 0.0, 0.25, and 0.5, which corresponded to exogeneity, moderate endogeneity, and high endogeneity, respectively. Similarly, the strength of the IVs, *κ*, was given values 0.01, 0.25, and 0.5, which is interpreted as weak IVs, moderately informative IVs, and strong IVs.

### 4.2 Results of the simulations

**Figure 2:**
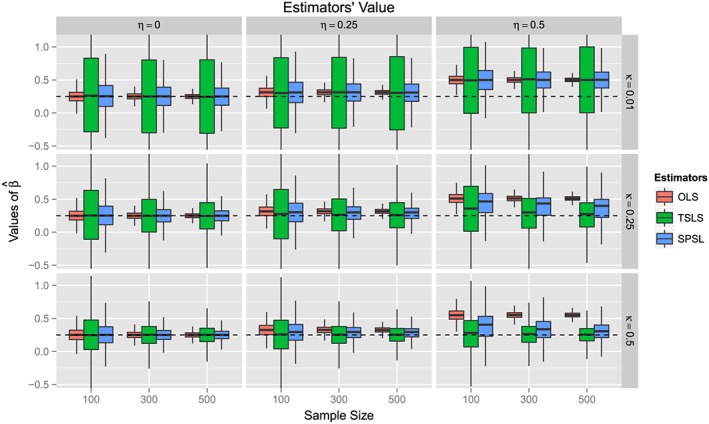
Approximate Monte Carlo distributions of the estimators' values under three different levels of confounding, $\eta =C\text{or}(S_{i},U_{i})$, and for three different levels of instrument's strength, $\kappa =C\text{or}(S_{i},B_{i}+R_{i}+R_{i}B_{i})$. In each panel, the sample size varies between n=100 and n=500. We here compare the ordinary least squares (OLS), two‐stage least squares (TSLS), and semi‐parametric Stein‐like (SPSL) estimators with respect to the true parameter β_S_=1/4, whose value is indicated by a dashed line. These simulations are based on 10^5^ iterations for each scenario.

The behavior of the SPSL was found to be mainly controlled by the strength of the instruments. When the instruments were strongly correlated with the predictor *S*, such that $\kappa =C\text{or}(S_{i},B_{i}+R_{i}+R_{i}B_{i})$ was large, the values of the SPSL estimator were close to the ones of the TSLS estimators, as can be observed in the last row of Figure 2. By contrast, when the instruments were weak, such that *κ* was small, the values of the SPSL estimator were closer to the ones of the OLS estimator, as can be seen in the first row of Figure 2.

**Figure 3:**
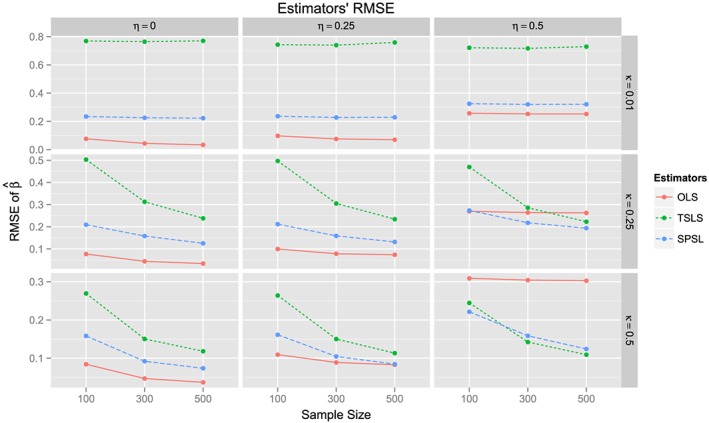
Monte Carlo estimates of the root mean squared errors (RMSEs) of the three estimators of interest under the simulation scenarios described in Figure 2. As predicted, the RMSE of the proposed semi‐parametric Stein‐like (SPSL) method strikes a trade‐off between its two constituent estimators. Indeed, under small η, the SPSL's RMSE approaches the RMSE of the ordinary least squares (OLS) estimator, whereas under large κ, it approaches the RMSE of the two‐stage least squares (TSLS) estimator. Note that the y‐scales of the row panels differ, depending on the value of κ.

When the true shrinkage is known, the SPSL is superior in quadratic risk to the OLS and TSLS. These Monte Carlo simulations appear to support a partial analog of this result when *α* is evaluated from the data. Indeed, Figure 3 shows that the MSE of the OLS estimator tends to be smaller than the MSE of the SPSL estimator, when no confounding is present, thereby showing that the SPSL's risk is not always superior to the risk of the OLS, when *α* is estimated from the data. On the other hand, one can observe from Figure 3 that the Monte Carlo MSE of the SPSL estimator is smaller than or equal to the one of the TSLS estimator under all considered scenarios, which justifies favoring the SPSL estimator over the TSLS estimator.

## 5 Dose–response analyses

In our data set, missing data were handled using multivariate imputation by chained equations (MICE), as implemented by 21 on the R platform. Multiple imputation produces valid inference, provided that (i) the assumed missing value mechanism is MAR; (ii) the relevant variables predicting missing values are included in the imputation; and (iii) the parameters in question are estimated using maximum likelihood. Note that, in the case of SoCRATES, the putative endogeneity of *S* does not conflict with the MAR assumptions, because multiple imputation solely requires observed variables, but not latent variables such as counterfactuals, to be predictive of the missingness in the outcome.

The estimators of the regression parameters described in the previous sections can be viewed as maximum likelihood estimators under the assumption of normality. The variables included in the imputation model, consisted of all the covariates, and the post‐randomization variables, such as therapy sessions and therapeutic alliance, as suggested by 22. The SEs for the resulting estimators were then constructed in a conventional way, using Rubin's rule. That is, given a *k*‐dimensional target estimator, ***β*** ^*†*^, the pooled SE for that estimator after imputing the different missing data points is given for every element $\beta_{j}^{†}$ of the vector ***β*** ^*†*^, with *j*=1,…,*k*, by the following formula:

$$\text{se}\left(\beta_{j}^{†}|D_{\text{obs}}\right):=\left(\right.\frac{1}{I}\overset{I}{\underset{i=1}{\sum }}\frac{\hat{V\text{ar}}\left(\beta_{j}^{†}|D_{i,\text{miss}},D_{\text{obs}}\right)}{n}+\frac{I+1}{I(I-1)}\overset{I}{\underset{i=1}{\sum }}\left(\beta_{ji}^{†}-{\bar{\beta }}_{j}^{†}\right){)}^{1/2},$$

where **D**_obs_ represents the entire observed data set; **D**_*i*,miss_ denotes the i*th* imputed data set, with *i*=1,…,*I*, *I* being the total number of imputations; and ${\bar{\beta }}_{j}^{†}$ is the average of the *I* estimated parameters, $\beta_{ji}^{†}$'s, which are based on each imputed data set. Finally, $\hat{V\text{ar}}(\beta_{j}^{†}|D_{i,\text{miss}},D_{\text{obs}})$ denotes the empirical variance of $\beta_{j}^{†}$ based on the i*th* imputed data set.

**Figure 4:**
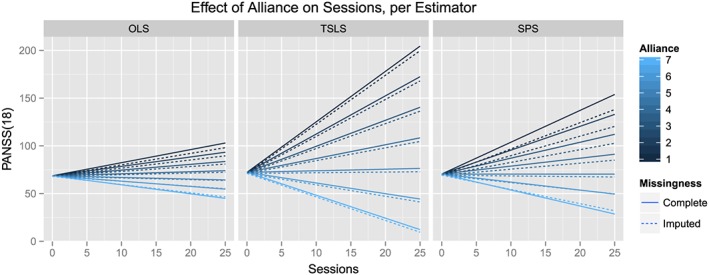
Effect of the number of sessions on PANSS(18), in the Study of Cognitive Re‐alignment Theory in Early Schizophrenia data set modified by alliance, using parameter estimates based on complete case analysis and after applying multiple imputation with chained equations, denoted by Complete and Imputed, respectively, and for the three different estimators. Therapeutic alliance as measured by California Therapeutic Alliance Scale has here been relabelled, such that minimal alliance is coded with one. It can be observed that the number of sessions of therapy (measured on the x‐axis) becomes detrimental to the number and severity of the symptoms (higher PANSS scores at 18 months, on the y‐axis), when therapeutic alliance diminishes (darker blue lines, denoting lower alliance).

We have here replicated and extended some of the results reported in table 3 of 3, for the analysis of the SoCRATES data set. We are fitting the linear model described in equations ((2)) and ((3)). In Table 1 of the present paper, we have compared the performances of the OLS and TSLS estimators with the ones of the SPSL estimator. An increase in the number of sessions that the subjects attended yielded a substantial decrease in psychotic symptoms reported by the subjects in the study, when a maximum level of alliance with the therapist was achieved (i.e., rescaled CALPAS score of zero). Importantly, this study evaluated the impact of therapeutic alliance on the effect of the number of sessions on outcome. This interaction is graphically illustrated in Figure 4. As expected, the greater was the self‐reported alliance with the therapist, the larger was the benefit of an extra session, as highlighted by a previous analysis of the same data set 15, 16. These results also show that attending extra sessions under poor therapeutic alliance has a detrimental effect on outcome. This interaction effect is captured by the estimate of the coefficient of the alliance × session product, ${\bar{\beta }}_{SA}=-0.83$(SE=0.27) for the SPSL estimator, thereby suggesting that the benefit of an extra session diminishes the score of PANSS(18) by 0.71 points, for every unit reduction in therapeutic alliance.

**Table 1:** Dose–response re‐analysis of the SoCRATES data set from 23.

Predictors	OLS	TSLS	SPSL
Complete cases a:		
Session	−0.95 (0.21)	−2.40 (0.65)	−1.68 (0.42)
Session× alliance	−0.39 (0.11)	−1.28 (0.45)	−0.83 (0.27)
PANSS(0)	0.38 (0.09)	0.39 (0.10)	0.39 (0.09)
Years of education	−1.11 (0.48)	−0.99 (0.60)	−1.05 (0.52)
Log DUP^b^	2.33 (2.63)	−0.20 (3.23)	1.06 (2.88)
Center 2	4.32 (3.92)	−1.22 (4.99)	1.55 (4.01)
Center 3	−11.96 (2.75)	−16.32 (3.59)	−14.15 (3.06)
SPSL $\hat{\alpha }$ c	–	–	0.50
Imputed missing cases d:		
Session	−0.90 (0.23)	−2.51 (0.86)	−1.88 (0.62)
Session× alliance	−0.35 (0.11)	−1.27 (0.52)	−0.91 (0.38)
PANSS(0)	0.36 (0.09)	0.37 (0.10)	0.37 (0.09)
Years of education	−1.17 (0.52)	−0.84 (0.66)	−0.97 (0.51)
Log DUP^b^	2.13 (2.38)	0.34 (3.08)	1.04 (2.66)
Center 2	5.02 (3.51)	0.36 (5.00)	2.18 (4.16)
Center 3	−11.43 (3.25)	−16.11 (4.78)	−14.28 (3.32)
SPSL $\hat{\alpha }$ c	–	–	0.61

SoCRATES, Study of Cognitive Re‐alignment Theory in Early Schizophrenia; OLS, ordinary least squares; TSLS, two‐stage least squares; SPSL, semi‐parametric Stein‐like; PANSS, Positive And Negative Syndrome Scale; SE, standard error; MICE, multiple imputation with chained equations.

Estimates for all predictors are reported, with bootstrapped standard errors in parentheses.

a Complete cases, for whom PANSS at month 18 was available, *n*=153.

b DUP here stands for duration of untreated psychosis in years.

c The estimated shrinkage used in the computation of the SPSL estimator. The SE for the SPSL estimator is based on 1000 bootstrap iterations.

d Missing data points were imputed using MICE with 100 imputations, thereby producing a data set with *n*=207 subjects.

For the complete cases, the values of the SPSL estimates were found to be bounded by the ones of the OLS and TSLS estimates. For the effect of sessions and for the interaction term between sessions and alliance, the values of the OLS estimator markedly differed from the ones of the TSLS estimator. Moreover, the TSLS estimators for these parameters exhibited greater SEs. Consequently, the SPSL estimator struck a balance between these two estimates, both in terms of its value and in terms of its SE. The shrinkage parameter, $\hat{\alpha }$, for both the complete and imputed data sets was close to 1/2, albeit it was found to favor the TSLS estimator for the imputed cases. We have here quantified the predictive power of the IVs by comparing the first‐stage models for the *S*_*i*_'s and the *S*_*i*_ *A*_*i*_'s, with and without the IVs. For the complete data set, the *F*‐statistics for the part of the model predicting the number of sessions was (*F*=227.24,df_1_=5,df_2_=143), and similarly for the *S*_*i*_ *A*_*i*_ term (*F*=33.18,df_1_=5,df_2_=143). *F*‐statistics were also computed after imputation of the missing data and provided similar results. Thus, these results suggested that there was no weak instrument bias in the TSLS estimator.

## 6 Discussion

In this paper, we have applied a method originally proposed by 8 for constructing Stein‐like estimators based on IV models, to a mental health trial. The re‐analysis of the trial described in this paper has demonstrated that the SPSL estimator should be incorporated in the toolbox of the researchers in this discipline.

The use of IV methods to analyze dose–response relationships – and consequently the use of the SPSL estimator for such problems – relies on effect homogeneity within relevant subpopulations, such as those defined by process variables, as described in the paper at hand. Our simulation studies also assume that treatment effect homogeneity holds across these subpopulations.

In this paper, we have concentrated on an application of the SPSL to dose–response models, with special emphasis on treatment effect modification by the number of sessions a patient actually attends. Naturally, this type of models could be applied to other situations, in which the effect of treatment is modified by other properties of treatment, such as qualitative differences in the delivery of therapy, for instance, 24. Furthermore, a topic for future research is the applicability of the SPSL to other trial‐related questions in which IV methods have been used, such as in causal mediation analysis.

In this paper, we have made three types of assumptions, which have been referred to as the causal, OLS and TSLS assumptions. The plausibility of these requirements in the context of the SoCRATES data set, can be justified as follows. Firstly, consider some of the main causal assumptions, such as assumption (C2), which states that we are fitting a linear dose–response model. Given the absence of any further information about the relationship between the dose and the response, such linear assumptions can be regarded as parsimonious. By contrast, if, in a different application, we had gathered more information about the existence of a nonlinear relationship between the dose and the response, it would then be possible to fit such data using polynomial regression. Moreover, for causal assumption (C3), observe that offer of treatment by itself has no effect on the outcome. Hence, it is reasonable to assume that the exclusion criterion assumption holds in our setting.

Secondly, for the assumptions pertaining to the OLS and TSLS estimation frameworks, it is reasonable to make standard assumptions, such as (OLS‐1) and (OLS‐2), about the homoscedasticity of the error terms and the identifiability of the predictors used in our model, because there was no evidence of substantial multicollinearity in the data at hand. A similar justification can be made for assumptions (TSLS‐2) and (TSLS‐3), regarding the identifiability of the TSLS estimator. Moreover, albeit the exogeneity of the instruments, stated in assumption (TSLS‐1), encompasses several sub‐assumptions owing to the fact that we are using a large number of instruments, it should be emphasized that all of these sub‐assumptions are, in fact, related. Indeed, we have here constructed a set of instruments, by interacting the baseline variables with the experimental factor. Other authors have made identical assumptions, when constructing relevant instruments for the SoCRATES data set 4, 6.

It is of special interest to consider the behavior of the SPSL estimator, when some of our assumptions fail to be satisfied. We here focus on the OLS and TSLS assumptions and evaluate how their violations may impact on the behavior of the combined Stein‐like estimator. Consider, for instance, condition (OLS‐2), which requires $X^{\prime }X$ to be identified, such that the expectation, $E[X^{\prime }X]$, is full‐rank. If such an assumption were to fail (or would be close to failure), then the condition number of the matrix, $E[X^{\prime }X]$, would be very high and the determinant of its inverse would be very large, thereby yielding a large OLS variance. Therefore, everything else being equal, the failure of (OLS‐2) would be likely to put the OLS at a disadvantage, in comparison with the TSLS.

A similar argument can be made, when considering a violation of (TSLS‐3). This assumption requires the matrices, $E[Z^{\prime }Z]$ and $E[Z^{\prime }X]$, to be full‐rank for the TSLS estimator to be identified. If this condition were to fail, the resulting TSLS variance would be unduly high. In this case, provided that the remaining assumptions would remain satisfied, a failure of (TSLS‐3) would then lead to the SPSL estimator being favored by the OLS. Moreover, the TSLS may also suffer, if condition (TSLS‐4) were to fail. This assumption requires that the instruments, **Z**, are relevant, in the sense that they should be correlated with the exogenous variables, **X**. If such an assumption were to be violated, this would put the TSLS at a disadvantage with respect to its counterpart, because the instruments would solely contribute to the TSLS estimator by inflating its variance, thereby rendering the OLS comparatively more efficient.

For finite *n*, the moments of the TSLS estimator and other *k*‐class estimators need not exist, as demonstrated by 25. It is common in such situations to assume that at least three instruments are present. This condition ensures that the first two moments of the estimators under scrutiny exist. Such an assumption is critical to the construction of the SPSL estimator, because the first two moments of the OLS and TSLS estimators are needed to compute the empirical estimator of the MSE. However, note that, asymptotically, all such moments exist. Thus, from an asymptotic perspective, this strategy can be applied to any number of instruments. Indeed, irrespective of the number of instruments used, every SPSL estimator is guaranteed to be asymptotically consistent. In fact, similar arguments are used to justify the use of most IV models, because such models tend to be only asymptotically identifiable.

The SPSL framework could be extended by allowing for a selection of certain parameters of interest in the vector, $\bar{\beta }(\alpha )$. Currently, the MSE criterion is minimized in a *global* fashion, for all the elements of the SPSL estimator. However, as we have seen with the re‐analysis, this type of global optimality can lead to counterintuitive results, because the values and SEs of some of the individual elements of the SPSL vector need not be comprised between the ones of the corresponding entries in the OLS and TSLS vectors. This issue could be addressed by selecting the SPSL estimator that *locally* minimizes the MSE for a subset of parameters of interest. Algebraically, this could be achieved by pre‐multiplying the MSE in order to select the subset of parameters of interest. For instance, one may be solely interested in finding the optimal SPSL estimator with respect to the number of sessions.

Note also that the OLS and TSLS estimators could be replaced by other candidate estimators when one estimator removes the bias at the cost of variance inflation, such as the jackknife IV estimator, for instance, introduced by 9. A combined estimator could be constructed in a similar fashion and would be likely to display comparable asymptotic properties. Other such estimators could be straightforwardly accommodated within the SPSL framework by estimating the MSE of the resulting combined estimator using the bootstrap. The rates of convergence of the asymptotic convergence of such combined estimators could also be studied by exploiting classical results in probability, such as the Berry–Esseen theorem. In addition, given the fact that the SPSL depends on the unbiasedness of the TSLS, it follows that the sensitivity of the SPSL to the linear assumptions made in Section 2.3 could be evaluated using the same type of sensitivity analysis used for the TSLS [e.g., 26].

The SPSL estimator therefore provides a general framework for striking a trade‐off between a biased but efficient estimator and an unbiased but inefficient estimator. Hence, one may consider how such a framework could be extended to other modeling strategies, such as fixed and random effects estimators for longitudinal data [see 14, for instance]. Similarly, this method could also be extended to combine competing estimators for measurement models. Observe that the estimators utilized to produce the SPSL estimator do not need to share the same data. Indeed, when constructing a combination of the OLS and TSLS estimators, only the TSLS estimator relies on the instrument, *Z*. In particular, note that the central limit theorem described by 19 could also enable the construction of statistical tests for evaluating whether or not the values of individual parameters are statistically significant. This would complete the development of the SPSL inferential framework.

## Appendix A Correspondence with linear model

### A.1

We here show that our parameters of interest represent the causal effects of specific explanatory variables in a linear model for the continuous outcome *Y*. We can write

$$\begin{array}{ccc} Y & =RY(R=1)+(1-R)Y(R=0) & \\ & =R\{Y(R=0)+\beta_{S}S(1)+\beta_{SA}S(1)A(1)+\nu \}+(1-R)Y(R=0)\\ & =Y(R=0)+\beta_{S}RS(1)+\beta_{SA}RS(1)A(1)+R\nu , \end{array}$$

utilizing the linear model assumption, (C2), for *Y*(*R*=1). Then,

$$Y(R=0)+\beta_{S}RS(1)+\beta_{SA}RS(1)A(1)+R\nu =Y(R=0)+\beta_{S}S+\beta_{SA}SA+R\nu ,$$

using the consequences of the no contamination assumption, (C1). Moreover,

$$Y(R=0)+\beta_{S}S+\beta_{SA}SA+R\nu =\mu_{1}+\beta_{B}B+\overset{k}{\underset{j=1}{\sum }}\beta_{j}X_{j}+\tau +\beta_{S}S+\beta_{SA}SA+R\nu ,$$

after invoking the linear model assumption, (C2), for *Y*(*R*=0).

Defining a combined error term, *ε*:=*τ*+*R* *ν*, we arrive at the following linear model equation for the observed outcome,

$$Y=\mu_{1}+\beta_{B}B+\overset{k}{\underset{j=1}{\sum }}\beta_{j}X_{j}+\beta_{S}S+\beta_{SA}SA+\varepsilon .$$

The derivation of this model equation only requires assumptions (C1) and (C2). The assumptions pertaining to the residual terms in (C3) and (C4), as well as exchangeability in (C5), ensure that E[ε|R=r]=0. That is, the expectation of *Y* is affected by treatment offer only via the observed number of sessions and alliance. Specifically, because of the exchangeability of random treatment offer assumed in (C5), we have

E[ε|R=r]=E[τ|R=r]+E[Rν|R=r]=E[Rν|R=r].

Furthermore, E[Rν|R=0]=0, and

$$\begin{array}{ccc} E[R\nu |R=1] & =E[\nu |R=1] & \\ & =P[S=0]E[\nu |R=1,S=0]+\underset{s>0,a}{\sum }P[S=s,A=a]E[\nu |R=1,S=s,A=a]\\ & =P[S=0]E[\nu |S(1)=0,R=1]+\underset{s>0,a}{\sum }P[S=s,A=a]E[\nu |S(1)A(1)=sa,S(1)=s], \end{array}$$

because of the no contamination assumption, (C1). Finally, using assumptions (C3) and (C4), the aforementioned equation can be rewritten as follows:

$$\begin{array}{c} P[S=0]E[\nu |S(1)=0,R=1]+\underset{s>0,a}{\sum }P[S=s,A=a]E[\nu |S(1)=s,A(1)=a]=0, \end{array}$$

and therefore E[ε|R]=E[ε].

## Appendix B Proofs of propositions

### B.1

B.1 Proof of Proposition 1

As mentioned in the text, we here assume that $\text{rank}(E[Z^{\prime }Z])=l,\text{rank}(E[Z^{\prime }X])=k$, and $\text{rank}(X^{\prime }X)=k$ hold. The proof of 1(i) immediately follows from the definition of the empirical bias in the body of the paper, which implies that the empirical bias of the TSLS estimator is identically zero, for every realization. For the proof of 1(ii), one needs to show that for every pair of matrices **X** and $\hat{X}:=H_{z}X$, we have $X^{\prime }X≽{\hat{X}}^{\prime }\hat{X}$. This can be conducted in two steps. Firstly, observe that we have the following equivalence because of the symmetry of **H**_*z*_:

(B1) $${\hat{X}}^{\prime }X={\left(H_{z}X\right)}^{\prime }X=X^{\prime }H_{z}X=X^{\prime }\hat{X}.$$

Secondly, the inner product of $\hat{X}$ can also be simplified using the idempotency of **H**_*z*_, such that

(B2) $$X^{\prime }\hat{X}=X^{\prime }H_{z}X=X^{\prime }H_{z}H_{z}X={\hat{X}}^{\prime }\hat{X}.$$

Then, expanding the dot product of $X-\hat{X}$ and applying equalities ((B1)) and ((B2)), we obtain

$${\left(X-\hat{X}\right)}^{\prime }(X-\hat{X})=X^{\prime }X-2X^{\prime }\hat{X}+{\hat{X}}^{\prime }\hat{X}=X^{\prime }X-{\hat{X}}^{\prime }\hat{X}.$$

Observe that the dot product, ${\left(X-\hat{X}\right)}^{\prime }(X-\hat{X})$, is a Gram matrix, and therefore, it is necessarily positive semi‐definite. Consequently, this implies that $X^{\prime }X-{\hat{X}}^{\prime }\hat{X}={\left(X-\hat{X}\right)}^{\prime }(X-\hat{X})≽0$, and hence $X^{\prime }X≽{\hat{X}}^{\prime }\hat{X}$, by the definition of the positive semi‐definite order, and moreover ${\left(X^{\prime }X\right)}^{-1}≼{\left({\hat{X}}^{\prime }\hat{X}\right)}^{-1}$, because these matrices were assumed to be invertible. Next, observe that the estimates of the error variances under the OLS and TSLS estimation procedures are defined as

$$(n-k){\tilde{\sigma }}^{2}:={\left(y-X\tilde{\beta }\right)}^{\prime }(y-X\tilde{\beta })⩽{\left(y-X\hat{\beta }\right)}^{\prime }(y-X\hat{\beta })=:(n-k){\hat{\sigma }}^{2},$$

where the inequality follows from the optimality of the OLS, and therefore, ${\tilde{\sigma }}^{2}{\left(X^{\prime }X\right)}^{-1}≼\;{\hat{\sigma }}^{2}{\left({\hat{X}}^{\prime }\hat{X}\right)}^{-1}$, after combining with the aforementioned inequality for $X^{\prime }X$ and ${\hat{X}}^{\prime }\hat{X}$, as required.

B.2. Proof of Proposition 2

The optimal value of *α* can be determined after a minimization of the criterion of interest, which will be denoted by $f(\alpha ):=\text{MSE}(\bar{\beta }(\alpha ))$. For expediency, we will expand this criterion as was carried out in equation ((6)) of the main body of the paper, such that

$$\text{tr}\;f(\alpha )=\text{tr}(\alpha^{2}M_{1}+2\alpha (1-\alpha )C+{\left(1-\alpha \right)}^{2}M_{2}),$$

with $M_{1}:=\text{MSE}(\hat{\beta }),C:=\text{CSE}(\hat{\beta },\tilde{\beta })$, and $M_{2}:=\text{MSE}(\tilde{\beta })$ and where recall that $\hat{\beta }$ and $\tilde{\beta }$ denote the TSLS and OLS estimators, respectively. Expanding the aforementioned expression for *f*(*α*) and taking the first derivative with respect to *α*,

$$\frac{\partial f(\alpha )}{\partial \alpha }=\frac{\partial }{\partial \alpha }\left(\right.\alpha^{2}M_{1}+2\alpha C-2\alpha^{2}C+(1-2\alpha +\alpha^{2})M_{2}),$$

which simplifies to

$$\frac{\partial f(\alpha )}{\partial \alpha }=2\alpha M_{1}+2C-4\alpha C+2\alpha M_{2}-2M_{2}.$$

Collecting these terms with respect to *α*, this produces *∂* *f*(*α*)/*∂* *α*=2*α*(*M*_2_−2*C*+*M*_1_)−2(*M*_2_−*C*). Moreover, because the derivative is a linear operator, it commutes with the trace, and we obtain

$$\text{tr}(\partial f/\partial \alpha )=2\alpha \text{tr}(M_{2}-2C+M_{1})-2\text{tr}(M_{2}-C).$$

Setting this expression to zero yields *α*:=tr(*M*_2_−*C*)/tr(*M*_2_−2*C*+*M*_1_), as required. Naturally, this minimization holds for every choice of *n*, thereby proving the first part of Proposition 2.

In addition, one can show that this minimizer is unique by performing a second derivative test, such that we obtain

(B3) $$\text{tr}(\partial^{2}f/\partial \alpha^{2})=2\text{tr}(M_{1}-2C+M_{2}).$$

Because by assumption, the random vectors, $\hat{\beta }$ and $\tilde{\beta }$, are element‐wise squared‐integrable, the components, $E[{\left({\hat{\beta }}_{j}-\beta_{j}\right)}^{2}]$, of *M*_1_ are finite for every *j*=1,…,*k*. Hence, using the linearity of the trace, the MSE of $\hat{\beta }$ can be treated as a sum of real numbers, thereby yielding the *L* ^2^‐norm on $R^{k}$, such that

$$\left(\right.\text{tr}\;\text{MSE}(\hat{\beta }){)}^{1/2}=\left(\right.\overset{k}{\underset{j=1}{\sum }}E\left[{\left({\hat{\beta }}_{j}-\beta_{j}\right)}^{2}\right]{)}^{1/2}=:||\hat{\beta }-\beta ||.$$

The latter quantity will be referred to as the (trace) root mean squared error of $\tilde{\beta }$.

By the same reasoning, it can be shown that *C* and *M*_2_ correspond to the inner product, $\left\langle \tilde{\beta }-\beta ,\hat{\beta }-\beta \right\rangle$, and the squared norm, $||\tilde{\beta }-\beta {\left|\right|}^{2}$ on $R^{k}$, respectively. Thus, equation ((B3)) can now be re‐expressed as follows:

$$\text{tr}(\partial^{2}f/\partial \alpha^{2})=2\left(\right.||\hat{\beta }-\beta {\left|\right|}^{2}-2\langle \hat{\beta }-\beta ,\tilde{\beta }-\beta \rangle +||\tilde{\beta }-\beta {\left|\right|}^{2}).$$

The Cauchy–Schwarz inequality can here be invoked to produce an upper bound on the cross term in the latter equation, $\left\langle \hat{\beta }-\beta ,\tilde{\beta }-\beta \rangle ⩽||\hat{\beta }-\beta ||\cdot ||\tilde{\beta }-\beta |\right|$. It then suffices to complete the square in order to obtain the following lower bound:

$$\text{tr}(\partial^{2}f/\partial \alpha^{2})⩾2\left(\right.||\hat{\beta }-\beta ||-||\tilde{\beta }-\beta ||{)}^{2}⩾0,$$

for every *n*, where equality only holds when the root mean squared errors of $\hat{\beta }$ and $\tilde{\beta }$ are identical, as required.

## Appendix C Simulation model

### C.1

We here describe the simulation model adopted in Section 5. Our objective in this Appendix is to justify our choice of parameters. The central difficulty is to express the error variances of the outcome variable and of the endogenous variable, in terms of the parameters in the model. Consider the model in equation ((8)). Firstly, the variance of the *S*_*i*_'s is given by

(C1) $$\begin{array}{ccc} V\text{ar}(S_{i}) & =\xi_{B}^{2}V\text{ar}(B_{i})+\xi_{R}^{2}V\text{ar}(R_{i})+\xi_{RB}^{2}V\text{ar}(R_{i}B_{i})+\eta^{2}V\text{ar}(U_{i})+\sigma_{\delta }^{2} & \\ & \;+2\xi_{B}\xi_{R}C\text{ov}(B_{i},R_{i})+2\xi_{B}\xi_{RB}C\text{ov}(B_{i},R_{i}B_{i})+2\xi_{B}\eta C\text{ov}(B_{i},U_{i})\\ & \;+2\xi_{R}\xi_{RB}C\text{ov}(R_{i},R_{i}B_{i})+2\xi_{R}\eta C\text{ov}(R_{i},U_{i})+2\xi_{RB}\eta C\text{ov}(R_{i}B_{i},U_{i}). \end{array}$$

The first line in the aforementioned equation can be simplified in the following manner: $\xi_{B}^{2}\sigma_{B}^{2}+\xi_{R}^{2}/4+\xi_{RB}^{2}\sigma_{B}^{2}/2+\eta^{2}+\sigma_{\delta }^{2}$, because by definition $\sigma_{R}^{2}=1/4$, and in addition, we also have $V\text{ar}(R_{i}B_{i})=V\text{ar}(R_{i})V\text{ar}(B_{i})+V\text{ar}(B_{i})E{\left(R_{i}\right)}^{2}=1/2\sigma_{B}^{2}$. Next, observe that $C\text{ov}(B_{i},R_{i})=C\text{ov}(R_{i},U_{i})=0$, because *R*_*i*_ is independent of both *B*_*i*_ and *U*_*i*_. Similarly, $C\text{ov}(B_{i},U_{i})=C\text{ov}(R_{i}B_{i},U_{i})=0$, because *U*_*i*_ is independent of both *B*_*i*_ and *R*_*i*_. Therefore, the remaining nonzero second‐order terms in equation ((C1)) are $C\text{ov}(B_{i},R_{i}B_{i})$ and $C\text{ov}(R_{i},R_{i}B_{i})$, and these are respectively given by $C\text{ov}(B_{i},R_{i}B_{i})=E(B_{i}R_{i}B_{i})-E(B_{i})E(R_{i}B_{i})=E(B_{i}^{2}R_{i})=1/2\sigma_{B}^{2}$, because the *B*_*i*_'s were assumed to be centered at zero, and using the fact that $C\text{ov}(R_{i},R_{i}B_{i})=E(R_{i}^{2}B_{i})-E(R_{i})E(R_{i}B_{i})=E(R_{i}^{2}B_{i})-E(R_{i})E(R_{i}B_{i})=0$, owing to $E(R_{i}^{2}B_{i})=E(R_{i}^{2})E(B_{i})=E(B_{i})$. Altogether, the variance of the *S*_*i*_'s can then be seen to reduce to the following expression:

(C2) $$V\text{ar}(S_{i})=\xi_{B}^{2}\sigma_{B}^{2}+\xi_{R}^{2}/4+\xi_{RB}\sigma_{B}^{2}(\xi_{B}+\xi_{RB}/2)+\eta^{2}+\sigma_{\delta }^{2}.$$

Secondly, the variance of the *Y*_*i*_'s, given by $V\text{ar}(Y_{i})=V\text{ar}(B_{i}\beta_{B}+S_{i}\beta_{S}+U_{i}\eta +\varepsilon_{i})$, can be decomposed as follows:

$$\begin{array}{c} V\text{ar}(Y_{i})=\beta_{B}^{2}\sigma_{B}^{2}+\beta_{S}^{2}V\text{ar}(S_{i})+\eta^{2}\sigma_{U}^{2}+\sigma_{\varepsilon }^{2}+2\beta_{B}\beta_{S}C\text{ov}(B_{i},S_{i})+2\beta_{B}\eta C\text{ov}(B_{i},U_{i})+2\beta_{S}\eta C\text{ov}(S_{i},U_{i}). \end{array}$$

The first covariance term, $C\text{ov}(B_{i},S_{i})$, in the aforementioned equation can be expanded as $C\text{ov}(B_{i},B_{i}\xi_{B}+R_{i}\xi_{R}+R_{i}B_{i}\xi_{RB}+U_{i}\eta +\delta_{i})$, which can be seen to reduce to $\sigma_{B}^{2}(\xi_{B}+\xi_{RB}/2)$, because *B*_*i*_,*R*_*i*_,*U*_*i*_, and *δ*_*i*_ are assumed to be pairwise independent. Similarly, one can verify that $C\text{ov}(B_{i},R_{i}B_{i})=E(B_{i}^{2}R_{i})=1/2\sigma_{B}^{2}$, for our choice of parametrization of *B*_*i*_ and *R*_*i*_. Moreover, we also have $C\text{ov}(S_{i},U_{i})=\eta$, because *U*_*i*_ is independent of all of the constituent variables of *S*_*i*_. Altogether, the variance of the outcome variable can therefore be expressed conditionally on the variance of *B*_*i*_, as follows:

(C3) $$V\text{ar}(Y_{i})=\beta_{B}^{2}\sigma_{B}^{2}+\beta_{S}^{2}+2\beta_{B}\beta_{S}\sigma_{B}^{2}(\xi_{B}+\xi_{RB}/2)+2\beta_{S}\eta^{2}+\eta^{2}+\sigma_{\varepsilon }^{2},$$

using the fact that $V\text{ar}(S_{i})$ is constrained to be 1 and where we have again used the fact that the *B*_*i*_'s and *U*_*i*_'s are pairwise independent.

From the aforementioned derivations, it can then be seen that the *degree of confounding* is standardized with respect to the parameter, *η*, such that the correlations between the predictor, *S*_*i*_, and the unmeasured confounder, *U*_*i*_, is given by $C\text{or}(S_{i},U_{i})=\eta$, as required. Moreover, we can also define the *instruments' strength*, *κ*, as a function of the parameters *ξ*_*B*_,*ξ*_*R*_, and *ξ*_*R**B*_. In order to do so, we need to guarantee that the variance of the summed instruments, *B*_*i*_+*R*_*i*_+*R*_*i*_ *B*_*i*_, is equal to unity. Using our previous derivations, one can verify that $V\text{ar}(B_{i}+R_{i}+R_{i}B_{i})=2.5\sigma_{B}^{2}+1/4$. Therefore, this variance can be set to 1, by choosing $\sigma_{B}^{2}:=0.3$. Then, we obtain $C\text{or}(S_{i},B_{i}+R_{i}+R_{i}B_{i})=\kappa$, where *κ*:=*ξ*_*B*_0.45+*ξ*_*R*_0.25+*ξ*_*R**B*_0.3, such that if we assume *ξ*_*B*_,*ξ*_*R*_, and *ξ*_*R**B*_ to be equal, it follows that this produces the further simplification *κ*=*ξ*_*B*_=*ξ*_*R*_=*ξ*_*R**B*_ because all the coefficients in *ξ*_*B*_0.45+*ξ*_*R*_0.25+*ξ*_*R**B*_0.3 sum to 1.

## References

[1] Dunn G , Maracy M , Dowrick C , Ayuso‐Mateos JL , Dalgard OS , Page H , Lehtinen V , Casey P , Wilkinson C , Vazquez‐Barquero JL . Estimating psychological treatment effects from a randomised controlled trial with both non‐compliance and loss to follow‐up. British Journal of Psychiatry 2003; 183(4):323–331. 14519610 10.1192/bjp.183.4.323

[2] Dunn G , Maracy M , Tomenson B . Estimating treatment effects from randomized clinical trials with noncompliance and loss to follow‐up: the role of instrumental variable methods. Statistical Methods in Medical Research 2005; 14(4):369–395. 16178138 10.1191/0962280205sm403oa

[3] Dunn G , Bentall R . Modelling treatment‐effect heterogeneity in randomized controlled trials of complex interventions. Statistics in medicine 2007; 26(26):4719–4745. 17476649 10.1002/sim.2891

[4] Maracy M , Dunn G . Estimating dose–response effects in psychological treatment trials: the role of instrumental variables. Statistical Methods in Medical Research 2011; 20:191–215. 19036909 10.1177/0962280208097243

[5] Small D . Mediation analysis without sequential ignorability: using baseline covariates interacted with random assignment as instrumental variables. Journal of Statistical Research 2012; 46(2):91–103. PMC4244702 25435642

[6] Ten Have TR , Joffe MM , Lynch KG , Brown GK , Maisto SA , Beck AT . Causal mediation analyses with rank preserving models. Biometrics 2007; 63(3):926–934. 17825022 10.1111/j.1541-0420.2007.00766.x

[7] Bound J , Jaeger DA , Baker RM . Problems with instrumental variables estimation when the correlation between the instruments and the endogeneous explanatory variable is weak. Journal of the American Statistical Association 1995; 90(430):443–450.

[8] Judge GG , Mittelhammer RC . A semiparametric basis for combining estimation problems under quadratic loss. Journal of the American Statistical Association 2004; 99(466):479–487.

[9] Angrist J , Imbens G , Krueger AB . Jackknife instrumental variables estimation, Technical Working Paper 172, National Bureau of Economic Research, Cambridge MA, 1995.

[10] Sawa T . Almost unbiased estimator in simultaneous equations systems. International Economic Review 1973; 14(1):97–106.

[11] Mittelhammer RC , Judge GG . Combining estimators to improve structural model estimation and inference under quadratic loss. Journal of econometrics 2005; 128(1):1–29.

[12] Judge GG , Mittelhammer RC . An Information Theoretic Approach to Econometrics. Cambridge University Press: Cambridge, UK, 2012.

[13] Rubin DB . Estimating causal effects of treatments in randomized and nonrandomized studies. Journal of educational Psychology 1974; 66(5):688–701.

[14] Wooldridge J . Econometric Analysis of Cross‐section and Panel Data. MIT press: London, 2002.

[15] Emsley R , Dunn G , White IR . Mediation and moderation of treatment effects in randomised controlled trials of complex interventions. Statistical Methods in Medical Research 2010; 19(3):237–270. 19608601 10.1177/0962280209105014

[16] Goldsmith L , Lewis S , Dunn G , Bentall R . Psychological treatments for early psychosis can be beneficial or harmful, depending on the therapeutic alliance: an instrumental variable analysis. Psychological medicine 2015; 45:1–9. 25805118 10.1017/S003329171500032X PMC4501302

[17] Hausman JA . Specification tests in econometrics. Econometrica 1978; 46(6):1251–1271.

[18] Judge G , Mittelhammer R . A Risk Superior Semiparametric Estimator for Over‐identified Linear Models, Advances in Econometrics, 2012, 237–255.

[19] Judge G , Mittelhammer R . A Minimum Mean Squared Error Semiparametric Combining Estimator, Advances in Econometrics, 2013, 55–85.

[20] Efron B , Morris C . Stein's estimation rule and its competitors: an empirical Bayes approach. Journal of the American Statistical Association 1973; 68(341):117–130.

[21] Buuren S , Groothuis‐Oudshoorn K . MICE: multivariate imputation by chained equations in R. Journal of statistical software 2011; 45(3):1–67.

[22] Barnard J , Frangakis CE , Hill JL , Rubin DB . Principal stratification approach to broken randomized experiments: a case study of school choice vouchers in New York City. Journal of the American Statistical Association 2003; 98(462):299–323.

[23] Lewis S , Tarrier N , Haddock G , Bentall R , Kinderman P , Kingdon D , Siddle R , Drake R , Everitt J , Leadley K . Randomised controlled trial of cognitive behavioural therapy in early schizophrenia: acute‐phase outcomes. The British journal of psychiatry 2002; 181(43):s91–s97. 10.1192/bjp.181.43.s91 12271807

[24] Dunn G , Fowler D , Rollinson R , Freeman D , Kuipers E , Smith B , Steel C , Onwumere J , Jolley S , Garety P . Effective elements of cognitive behaviour therapy for psychosis: results of a novel type of subgroup analysis based on principal stratification. Psychological medicine 2012; 42(05):1057–1068. 21939591 10.1017/S0033291711001954 PMC3315767

[25] Kinal TW . The existence of moments of *k*‐class estimators. Econometrica 1980; 48(1):241–249.

[26] Small DS . Sensitivity analysis for instrumental variables regression with overidentifying restrictions. Journal of the American Statistical Association 2007; 102(479):1049–1058.

